# Supplementary material for: Causal Relationship between Aging and Anorexia Nervosa: A White-Matter-Microstructure-Mediated Mendelian Randomization Analysis
Source: Biomedicines. 2024 Aug 16;12(8):1874. doi: 10.3390/biomedicines12081874 (PMC11351342; doi:10.3390/biomedicines12081874)
Supplement: Supplementary file 1 [file biomedicines-12-01874-s001.zip › Supplementary Figure/Figure S9/Leaveoneout plot of MR analysis for ubm-b-1913 to AN.pdf]

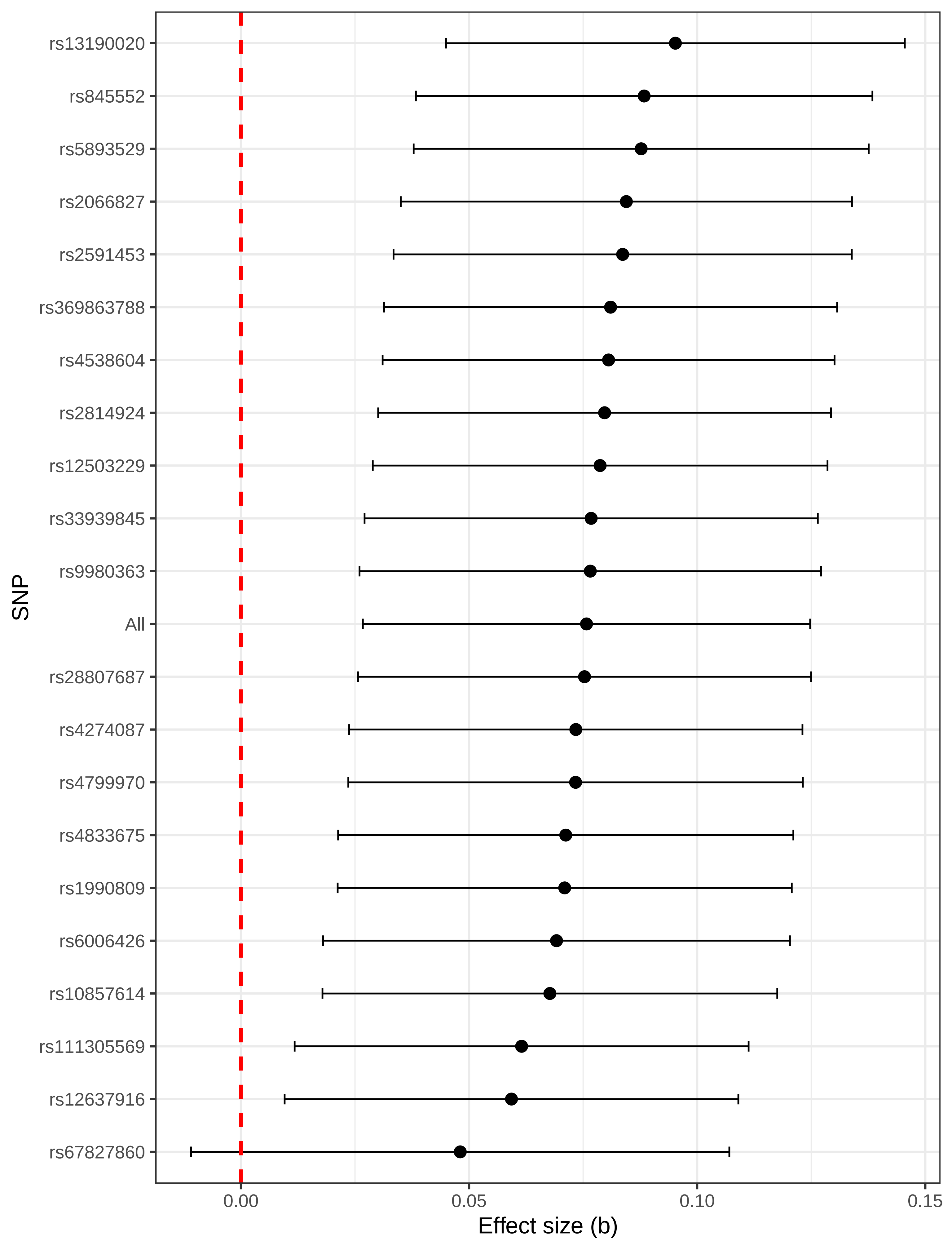

rs13190020
rs845552
rs5893529
rs2066827
rs2591453
rs369863788
rs4538604
rs2814924
rs12503229
rs33939845
rs9980363
All
rs28807687
rs4274087
rs4799970
rs4833675
rs1990809
rs6006426
rs10857614
rs111305569
rs12637916
rs67827860
SNP
0.00
0.05
0.10
0.15
Effect size (b)
